# Agmatine Administration Effects on Equine Gastric Ulceration and Lameness

**DOI:** 10.3390/jcm11247283

**Published:** 2022-12-08

**Authors:** Takashi Taguchi, Francisco J. Morales Yniguez, Catherine Takawira, Frank M. Andrews, Mandi J. Lopez

**Affiliations:** 1 Laboratory for Equine and Comparative Orthopedic Research, Department of Veterinary Clinical Sciences, School of Veterinary Medicine, Louisiana State University, Baton Rouge, LA 70803, USA; 2 Department of Veterinary Clinical Sciences, School of Veterinary Medicine, Louisiana State University, Baton Rouge, LA 70803, USA

**Keywords:** osteoarthritis, nonsteroidal, gait, pharmacokinetics, metabolomics

## Abstract

Osteoarthritis (OA) accounts for up to 60% of equine lameness. Agmatine, a decarboxylated arginine, may be a viable option for OA management, based on reports of its analgesic properties. Six adult thoroughbred horses, with lameness attributable to thoracic limb OA, received either daily oral phenylbutazone (6.6 mg/kg), agmatine sulfate (25 mg/kg) or a control for 30 days, with 21-day washout periods between treatments. Subjective lameness, thoracic limb ground reaction forces (GRF), plasma agmatine and agmatine metabolite levels were evaluated using an established rubric, a force platform, and mass spectrometry, respectively, before, during and after each treatment period. Gastric ulceration and plasma chemistries were evaluated before and after treatments. Braking GRFs were greater after 14 and 29 days of agmatine compared to phenylbutazone administration. After 14 days of phenylbutazone administration, vertical GRFs were greater than for agmatine or the control. Glandular mucosal ulcer scores were lower after agmatine than phenylbutazone administration. Agmatine plasma levels peaked between 30 and 60 min and were largely undetectable by 24 h after oral administration. In contrast, plasma citric acid levels increased throughout agmatine administration, representing a shift in the metabolomic profile. Agmatine may be a viable option to improve thoracic limb GRFs while reducing the risk of glandular gastric ulceration in horses with OA.

## 1. Introduction

Osteoarthritis (OA) accounts for over 60% of equine lameness in the United States, with an annual socioeconomic impact as high as $1 billion [1,2]. There are limited options to inhibit OA progression, and most have variable outcomes; hence, the primary treatment focus is often the alleviation of joint pain [3]. Non-steroidal anti-inflammatory drugs (NSAIDs) are routinely administered to manage pain from OA. Therapeutic levels are rarely achieved in plasma or synovial fluid with topical application [4], and systemic administration, especially long-term, increases risks of renal and gastrointestinal toxicity [5]. Alternatives to standard NSAIDs for alleviating OA discomfort may increase options for long-term use.

Pain associated with OA is multifaceted and has both nociceptive and neuropathic contributions that vary at different stages of joint degeneration [6,7]. Neuropathic pain, reported to predominate in the early stages, is often refractory to NSAIDs [8,9]. Macroscopic synovial-associated OA changes consisting of reduced peripheral nerve numbers and increased vascularization, both characteristics of neuropathic changes, suggest neuropathic elements of equine OA pain [10]. Equine hooves with pain from laminitis, which is also often refractory to NSAIDs, are also reported to develop neuropathic changes, including reduced unmyelinated and myelinated nerve fibers and upregulation of sensory nerve cell body neuronal injury markers [11]. The ability to alleviate equine neuropathic discomfort could significantly improve the quality of life for horses with OA. Additionally, neuropathic pain itself contributes to OA progression [12,13]. Hence, alleviation of neuropathic OA pain may benefit retired horses and extend the careers of working and pleasure horses.

Agmatine, a naturally occurring compound formed by arginine decarboxylation [14,15], is reported to have dose-dependent neuroprotective and anti-neuropathic effects, primarily via the modulation of α_2_-adrenergic and imidazoline receptors, combined with the antagonism of N-methyl-D-aspartate receptors and inhibition of inducible nitric oxide (NO) synthase [16,17,18,19,20,21,22,23,24,25]. Agmatine effects have been most frequently evaluated in rats for the treatment of conditions like thermal allodynia and hyperalgesia from local injury or systemic illness [24,26]. Agmatine administration was also recently reported to improve pelvic limb use in dogs with mild coxofemoral joint OA [27]. Furthermore, NO plays a central role in equine and human OA progression, so the inhibition of NO synthetase isoforms may slow joint degeneration [28,29]. Based on this information, agmatine may be a promising adjunct to current approaches for improved limb use in horses with OA.

Both systemic and local gastroprotective effects of agmatine are also reported, although most evidence is available from rat models of gastric injury. Specifically, intraperitoneal administration reportedly preserves mucosal integrity and reduces vascular congestion in a rat gastric ischemia–reperfusion model [30], and intracerebroventricular administration inhibits ethanol-induced gastric mucosal damage [31]. Additionally, polyamine metabolites of agmatine, including supermidine, supermine, and putrescine, have gastroprotective effects in rat models of ethanol-, aspirin-, and stress-induced gastric ulceration [32,33,34]. Putrescine, the primary metabolite of agmatine, is naturally produced by human intestinal microbiota, and agmatine administration increases the production of the metabolite; potentially augmenting inherent gastroprotection [32]. At present, there is limited direct evidence of an agmatine gastroprotective effect in horses. However, a compound that alleviates equine OA pain and has gastroprotective properties would be a promising addition to current therapeutic options.

Based on the existing information, three hypotheses were tested in this study: (1) Agmatine improves limb use in horses with naturally occurring thoracic limb OA, comparable to an established NSAID, phenylbutazone, and more than the control; (2) Equine gastric ulcer scores are greatest with phenylbutazone administration, followed by the control, and scores for both are greater than with agmatine administration; (3) Equine plasma agmatine levels increase following oral administration. To test the hypotheses, limb use and gastric ulceration were measured in horses with naturally occurring thoracic limb OA before, during and after the administration of agmatine, phenylbutazone, and the control. Additionally, plasma agmatine levels and primary metabolites were quantified at intervals during each treatment block.

## 2. Materials & Methods

### 2.1. Study Design

**Figure 1:**
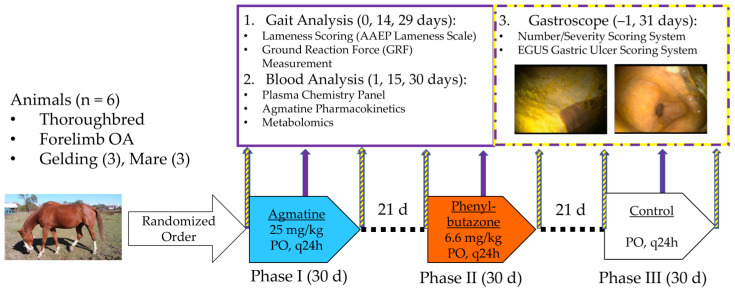
Three-phase, 1 randomized, controlled, crossover study design. Six adult thoroughbred horses with thoracic limb osteoarthritis (OA) received oral agmatine, phenylbutazone, or control in random order. Gait analysis and blood collection were performed before, during, and after each treatment phase, while gastroscopy was performed before and after. AAEP: American Association of Equine Practitioners; EGUS: equine gastric ulcer syndrome; PO: per os.

The study was a prospective, randomized, placebo-controlled, three-way crossover design (Figure 1). Baseline values for each treatment block were obtained according to an established timeline within 2 days before treatment, and they were timed to avoid influencing each other (i.e., gastroscopy sedation and gait measures). Specifically, baseline data were collected on day −1 for gastroscopy, day 0 for subjective and objective kinetic gait analysis, and day 1 at time 0 (immediately prior to administration of treatment) for plasma chemistry and agmatine level analysis. Outcome measures were normalized by individual horse baseline values to serve as internal controls and minimize interindividual variances and potential environmental effects. Horses received agmatine sulfate (25 mg/kg, PO: G-Agmatine^®^ brand, Gilad & Gilad LLC, Henderson, NV, USA), phenylbutazone (6.6 mg/kg, PO: Phenylbute^®^ Powder, Clipper LLC, St. Joseph, MO, USA), or control (Omolene^®^ 200, Purina Mills LLC, Gray Summit, MO, USA) for 30 days each in random order. Horses did not receive any medication during a 21-day washout period between treatments. Treatments, agmatine or phenylbutazone, were administered once daily from coded containers as a top dressing on commercially available concentrate feed that also served as the control (2.2 kg daily: Omolene^®^ 200). After each administration, it was confirmed that horses consumed more than 98% of the treatments within 10 min subjectively.

The kinetic gait data were obtained on days 0, 14, and 29, and blood samples were collected on days 1, 15, and 30 of the treatment blocks. Gastroscopy was performed one day prior to initiation and one day after the last treatment or control administration (days −1 and 31). The investigators who collected blood samples, recorded gait data, or quantified gastric ulceration were unaware of treatments until data collection, reduction, and analysis were complete.

All procedures were approved by the Institutional Animal Care and Use Committee (protocol #18-099) prior to the investigation initiation. Six adult horses were selected from an institutional research herd that are retired from any exercise or work regimens, based on the following inclusion criteria: (1) Thoroughbred; (2) Four–twenty-five years of age; (3) Mare or gelding; (3) 350–600 kg; (4) Radiographic evidence of bilateral thoracic limb OA; (5) Bilateral grade 1–3 thoracic limb lameness according to the American Association of Equine Practitioners (AAEP) lameness scale [35] (Table 1); (6) No signs of systemic illness or musculoskeletal trauma based on a physical exam; (7) A minimum of 3 months since inclusion in a prior investigation. All horses received a complete physical exam by a licensed veterinarian prior to the investigation and once daily during treatment blocks. Plasma aspartate aminotransferase (AST), alanine aminotransferase (ALT), alkaline phosphatase (ALP), glucose (GLU), blood urea nitrogen (BUN), and creatinine (CREAT) levels were measured before the first and after the last dose of all treatments. A professional farrier trimmed and leveled the hooves of horses selected for the study approximately 5 days prior to the beginning of each treatment phase. Horses were housed individually in 3.6 × 3.6 m^2^ stalls with concrete floors covered by rubber mats for 2–3 days prior to the first (days −2 to −1 of each phase) and after (days 32 to 33 of each phase) the last treatment administration of individual treatment blocks. Horses had ad libitum water in a 16 L bucket that was refreshed a minimum of two times per day and received mixed grass square-bale hay (1.5% body weight) twice daily on a set schedule. During the 21-day washout periods between treatment blocks, horses remained in pastures with their established herds and were allowed to exercise freely, but not according to a specific schedule or program.

### 2.2. Kinetic Gait Analysis

The same board-certified veterinary surgeon, unaware of the treatment, assigned all subjective lameness scores for individual thoracic limbs according to the AAEP lameness scale [36]. Each horse was observed in hand at a walk, a trot in a straight line, and lunging in clockwise and counterclockwise directions. The change in score from the baseline for the right and left thoracic limbs were combined to give a single value at all time points. Score change was calculated as ((score at each time point)–(score at baseline)).

The objective kinetic gait data were recorded as previously described [27,37,38]. Briefly, ground reaction forces (GRFs) were measured with a 90 × 90 cm^2^ force platform (Model # OR6-WP-1000, Advanced Mechanical Technology, Inc., Watertown, MA, USA) embedded in the center of a 40 m concrete runway. The surface of the force platform was the same color and texture as the runway. Horses were conditioned to trot over the force platform prior to the study initiation, and experienced handlers trotted horses for all trials. A series of five retroreflective photocell sensors (Mek92—PAD, Joslyn Clark Controls, Inc., Lancaster, PA, USA) were used to calculate trial velocity and acceleration. A trial was considered successful if a thoracic limb and the ipsilateral pelvic limb contacted the force platform at a velocity of 2.0 to 4.0 m/s and an acceleration of −1.0 to 1.0 m/s^2^. The velocity and acceleration ranges included a natural trot for all horses, who were allowed to move freely without interference from the handler. Trials were rejected if the hoof was not entirely on the force platform, was not straight on the platform, or was within 5 cm of the platform edge. A minimum of five successful trials were recorded for the left and right sides at all time points. Data logging was triggered by a force ≥ 50 N, and all trials were recorded at a rate of 1 × 10^3^ Hz and subsequently processed with commercially available software (Acquire v7.3, Sharon Software Inc., Dewitt, AR, USA).

The measured GRF values included craniocaudal (y plane) braking and propulsion and vertical (z plane) peak force and impulse [27]. For reporting purposes, the mean GRF value of the right and left thoracic limbs were combined to give a single value for individual horses for the five trials, with 30 values at each time point [38]. At each time point and for all GRF measures, the percentage change from the baseline was calculated as ((GRF_treatment_ − GRF_baseline_)/(GRF_baseline_)) × 100. The thoracic limb symmetry index (SI) was calculated as (mean thoracic limb GRF) − absolute value (right–left thoracic limb GRF))/(mean thoracic limb GRF).

### 2.3. Gastroscopy

The non-glandular and glandular gastric mucosa was examined with a video endoscope (3 m length × 10.4 mm diameter, Karl Storz, El Segundo, CA, USA), and images of ulcerated areas were documented with an integrated imaging system. To improve visualization of the stomach, food, but not water, was withheld for 16–18 h prior to examination when a muzzle was placed on each horse to prevent ingestion of shavings or other environmental material. Horses were sedated with xylazine (0.4 mg/kg IV, XylaMed™, Bimeda-MTC, Cambridge, ON, Canada), and, following the passage of the endoscope, the stomach was insufflated with air (Airhead 120V Hi-Pressure Air Pump, Kwik Tek, Inc., Denver, CO, USA) until rugae were no longer visible. The mucosal surfaces of the stomach were cleansed of mucus and debris with tap water flushed through the biopsy channel of the endoscope. The equine gastric ulcer syndrome (EGUS) system [39] and a validated rubric with two parts, ulcer number and ulcer severity [40] (Table 2 and Table 3) were used by a board-certified internist, with expertise using the rubrics and unaware of treatment blocks, to assign three scores: EGUS, ulcer number, and ulcer severity, for glandular mucosa and two scores, ulcer number and ulcer severity, for non-glandular mucosa. Score change, calculated as (day 31 score)–(baseline score), was used for analysis. Suction was used to remove insufflated gastric air at the end of the procedure.

### 2.4. Plasma Pharmacokinetic and Metabolomic Analysis

Plasma levels of agmatine and primary agmatine metabolites were measured in samples collected prior to, and 30, 60, 120 min and 5 and 24 h after agmatine consumption on the 1st, 15th, and 30th day of administration and after control consumption on the 15th day of administration. For blood collection, a 14 G × 5.25” catheter (MILA International, Inc., Florence, KY, USA) was placed in the left or right jugular vein and secured with #2–0 nylon suture (Ethilon™, Ethicon Inc., Raritan, NJ, USA) following aseptic preparation. A 20” extension set (ICU Medical Inc., San Clemente, CA, USA) and three-way stopcock (B. Braun Medical Inc., Melsungen, Germany) were attached to the catheter, and the system was flushed with 7 mL of heparinized saline (1 IU/mL) after each blood sample was drawn. Prior to sample collection, 5 mL of blood was aspirated through the system and discarded. Subsequently, 9 mL of blood was withdrawn and immediately distributed among 3 mL lithium heparin tubes (BD Vacutainer^®^, BD, Franklin Lakes, NJ, USA) that were inverted several times prior to being stored on ice for no more than 1 h. Following centrifugation (3.0 × 10^3^× *g*) for 30 min, the plasma supernatant was removed and placed in 1 mL cryovials (Nunc Cryotube Vials, Thermo Fisher Scientific, Waltham, MA, USA) that were stored at −80 °C. Samples were shipped on dry ice to a commercial vendor (West Coast Metabolomics Center, University of California, Davis, CA, USA) for the quantification of agmatine and agmatine metabolites.

On the day of analysis, plasma samples were thawed at room temperature and 30 µL transferred to a 1.5 mL Eppendorf tube. One mL of chilled 3:3:2 ACN: IPA: H_2_O was added to each tube. Tubes were vortexed for 10 s, shaken for 5 min at 4 °C on an orbital mixer (Thomas Scientific, Swedesboro, NJ, USA), and then centrifuged for 2 min at 1.4 × 10^4^× *g*. A total of 450 µL of supernatant was transferred to a new 1.5 mL tube, then dried via centrifugal vacuum concentrator (centrivap, Labconco, Kansas City, MO, USA). Dried samples were reconstituted in 100 µL 80:20 ACN: H_2_O, vortexed for 10 s, sonicated for 5 min, and then centrifuged for 2 min at 1.61 × 10^4^× *g*. A total of 90 µL of supernatant from each sample was transferred to an LC-MS vial for analysis. A standard curve and samples were injected onto a BEH amide 150 mm × 2.1 mm, 1.7 µm column connected to a TripleTOF 6600 mass spectrometer (SCIEX, Framingham, MA, USA) operating in positive mode. Extracted ion chromatograms of the target analyte, agmatine, were then used to determine peak areas for subsequent quantification.

The peak plasma concentration (Cmax) and the time to reach Cmax (Tmax) were determined [41]. The area under the agmatine plasma concentration versus time curve (AUC) was calculated using commercially available software (v 4.1.1 PK package in R software). Peak intensities of primary metabolites in each sample were normalized to the average peak intensities in pooled samples and analyzed using Metaboanalyst 5.0 (https://www.metaboanalyst.ca/ accessed on 15 October 2021). Heatmap, two-way ANOVA, and random forest analyses were performed using a time-series design with a one-factor of 4 levels.

### 2.5. Statistical Analysis

The normality of score changes for AAEP, EGUS, and the number and severity of the non-glandular and glandular mucosa ulcers, as well as plasma agmatine levels, Cmax, and Tmax, were evaluated with the Kolmogorov–Smirnov test. Score changes were compared to the baseline (score change 0) at each time point for each treatment as an indirect analysis of treatment effects using one-sample t-tests and Wilcoxon signed rank tests for parametric and nonparametric data, respectively. Additionally, baseline values for all outcome measures were compared among treatment blocks using one-way ANOVA. Repeated measures of two-way ANOVA were used to compare plasma agmatine levels with fixed effects of treatment (agmatine 1st, 15th, 30th day; control 15th day) and time (0, 30, 60, 120 min, 5, 24 h) as well as treatment × time interaction. Plasma agmatine Cmax, Tmax, and AUC were compared among treatments (agmatine 1st, 15th, 30th day; control 15th day) using one-way ANOVA. A linear mixed model was used to compare the fixed effects of treatment, time, and treatment × time interaction with the random effect of the horse on gait measures, gastric ulcer scores, and plasma chemistry levels. Tukey–HSD post hoc tests were performed when there were statistically significant differences in group means. Residual homogeneity and normality were examined by predicted and residual quantile plots, respectively. All analyses were performed using commercially available software (JMP, v14, SAS Institute Inc., Cary, NC, USA; Prism v7, GraphPad Software Inc., San Diego, CA, USA). Data are presented as least squares (LS) mean (mixed model) or mean (repeated measures two-way ANOVA) ± standard error of the mean (SEM). Significance was considered at *p* < 0.05.

## 3. Results

### 3.1. Horses

**Figure 2:**
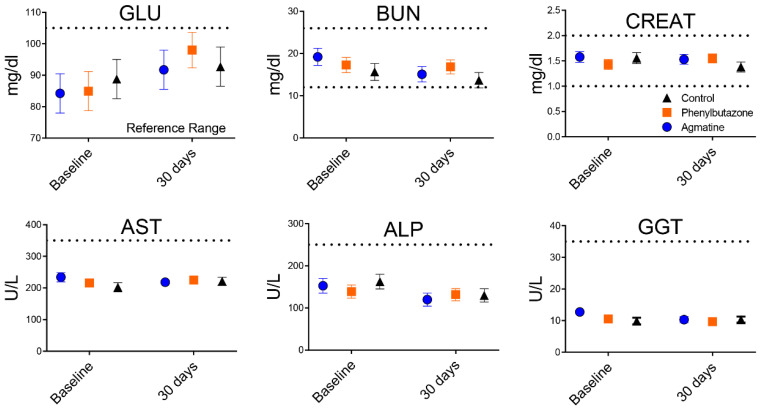
Plasma chemistry values before and after 30 days of oral agmatine (blue), phenylbutazone (orange), or control (black) administration. Normal reference ranges are indicated by dotted lines. GLU: glucose; BUN: blood urea nitrogen; CREAT: creatinine; AST: aspartate transaminase; ALT: alanine aminotransaminase; GGT: gamma-glutamyl transferase.

A total of 6 thoroughbred horses, three mares and three geldings, 12 ± 2.9 years of age (mean ± SEM, range 4–21 years) with a body weight of 487.9 ± 30 (range 378.6–580.9 kg) were used for this investigation. There were no detectable adverse effects during or after any treatment administration based on routine physical examination. Plasma GLU (*p* = 0.8166), BUN (*p* = 0.2795), CREAT (*p* = 0.5579), AST (*p* = 0.6053), ALP (*p* = 0.7688), and GGT (*p* = 0.2108) were not different among treatments within each time point or between time points within each treatment and remained within normal reference ranges throughout the study (Figure 2).

### 3.2. Gait Analysis

**Figure 3:**
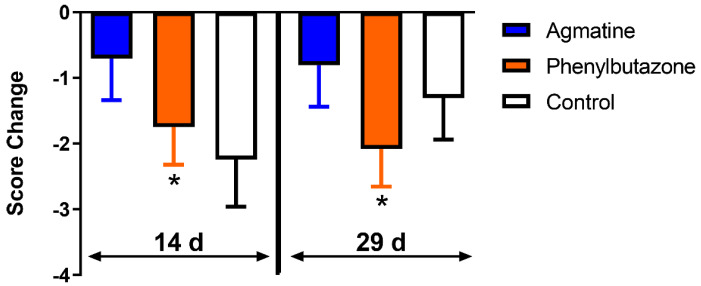
Changes in subjective AAEP Lameness Score (LS mean ± SEM). The AAEP lameness score changes after 14 (14 d) and 29 (29 d) days of oral agmatine (blue), phenylbutazone (orange) or control (white) administration relative to the baseline. Individual thoracic limb scores were combined to give a single score for each horse at all time points. Asterisks indicate a significant change (*p* < 0.05) from the baseline.

There were no significant differences in the baseline subjective lameness scores among treatments. Subjective lameness scores decreased after 14 and 29 days of phenylbutazone administration compared to the baseline. The change in subjective lameness was not significantly different among treatments at any time point (Figure 3 and Table 4). There were no treatment, time, or treatment and time interaction effects (Table 5).

**Figure 4:**
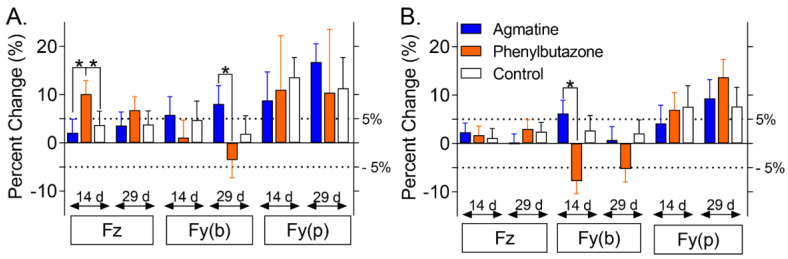
Percent change (LS mean ± SEM) in thoracic limb ground reaction forces over the baseline. Peak force (**A**) and impulse (**B**) in vertical (Fz) and craniocaudal braking (Fy(b)) and propulsion (Fy(p)) after 14 and 29 days of oral agmatine (blue), phenylbutazone (orange), or control (white) administration. Asterisks indicate differences among treatments within time points (*p* < 0.05). A 5% change from the baseline (dotted lines) is indicated.

The percentage increase in peak vertical force (Fz) after 14 days of phenylbutazone administration was higher than both agmatine (*p* < 0.0001) and the control (*p* = 0.0002), and the percentage increase in braking (Fy(b)) peak force after 29 days (*p* = 0.0028), as well as braking impulse after 14 days (*p* = 0.0083) of agmatine administration, was higher than after phenylbutazone administration at the same time points. (Figure 4). There were treatment effects of agmatine and phenylbutazone on peak force (Fz) as well as treatment and time interaction effects of phenylbutazone on peak force (Fz) and agmatine on peak force (Fy(p)), respectively (Table 6). Additionally, there were treatment effects of agmatine and phenylbutazone on impulse (Fy(b)) as well as treatment and time interaction effects of agmatine on impulse (Fz, Table 7). The symmetry index was not different for any kinetic measure among treatments at any time point or among time points for any treatment.

### 3.3. Gastroscopy

**Figure 5:**
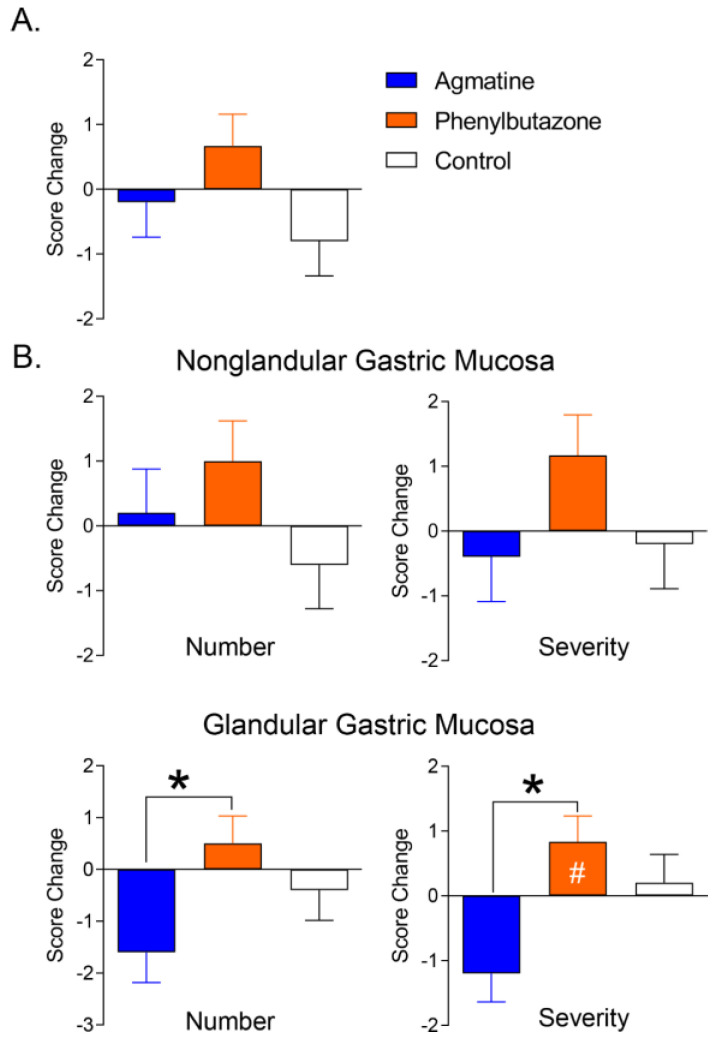
Changes in equine gastric ulcer syndrome (EGUS) scores (**A**) and number and severity of gastric non-glandular and glandular mucosa ulcer scores (**B**) between 1 day before and after 31 days of oral agmatine (blue), phenylbutazone (orange) or control (white) administration (LS mean ± SEM). Asterisks indicate significant differences among treatments (*p* < 0.05). A hash symbol indicates a significant change from the baseline.

There was no difference among treatments in the baseline in EGUS or number and severity scores. Glandular mucosa severity scores increased (*p* = 0.0422) from the baseline after phenylbutazone administration (Figure 5 and Table 8). Additionally, glandular mucosa ulcer severity (*p* = 0.0115) and number (*p* = 0.0472) were lower after agmatine versus phenylbutazone administration. There were treatment effects of agmatine (β = −1.1444, *p* = 0.0063) and phenylbutazone (β = 0.8889, *p* = 0.0204) on score changes in glandular number and severity (Table 9).

**Figure 6:**
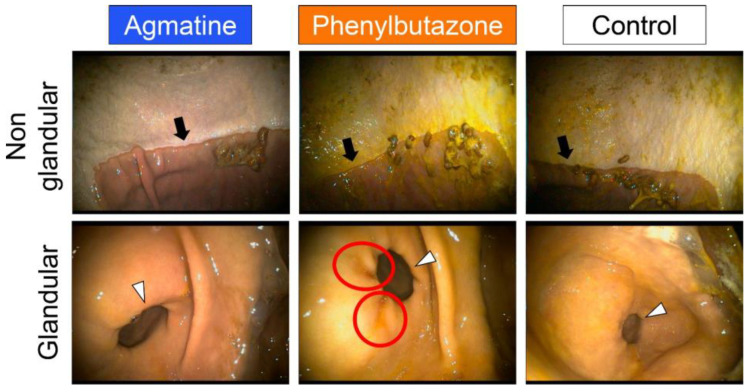
Representative gastroscopic images of equine non-glandular and glandular gastric mucosa after 30 days of oral agmatine (**left**), phenylbutazone (**middle**) or control (**right**) administration. Ulcers in the glandular region are indicated with red circles. The margo plicatus (black arrows) and pylorus (white arrowheads) are also visible.

Mild to moderate hyperkeratosis and bile staining of the glandular mucosa after phenylbutazone and control treatment were observed (Figure 6). Glandular mucosa ulceration was most evident after phenylbutazone administration. Following agmatine administration, there was no glandular mucosa hyperemia and minimal non-glandular mucosa hyperkeratosis.

### 3.4. Plasma Pharmacokinetic and Metabolomic Analysis

**Figure 7:**
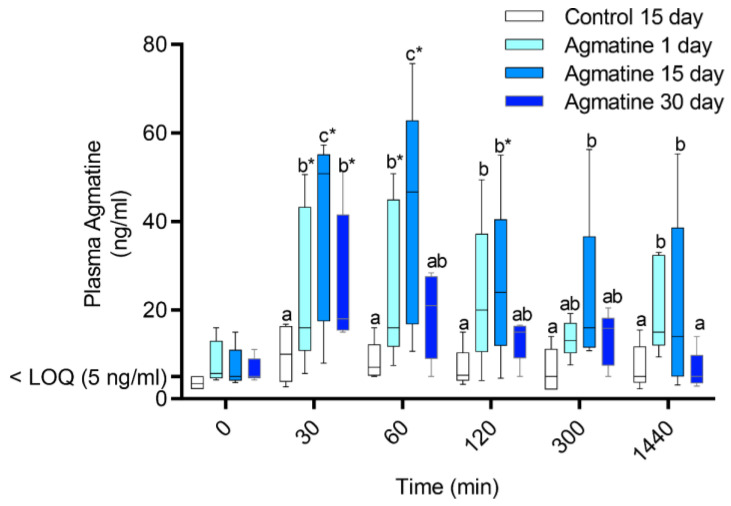
Equine plasma agmatine levels after 1 (light blue), 15 (blue), or 30 (dark blue) days of agmatine, or 15 days of control (white) oral administration. Distinct lowercase letters indicate differences among treatment/time cohorts (agmatine 1st, 15th, 30th day; control 15th day) within each time point (*p* < 0.05). Asterisks indicate differences between each time point and the baseline (time 0) within each treatment/time cohort. LOQ: limit of quantification.

All treatment (*p* = 0.0001), time (*p* = 0.0236), and treatment × time interaction (*p* = 0.0115) effects on plasma agmatine levels were significant. Plasma agmatine concentrations were higher 30 (*p* = 0.0269) and 60 (*p* = 0.0155) minutes after agmatine consumption compared to the baseline (0 min) on the first day. Similarly, they were higher 30 (*p* < 0.0001), 60 (*p* < 0.0001), and 120 (*p* = 0.0085) minutes after consumption on the 15th day and 30 min (*p* = 0.0042) after consumption on the 30th day compared to the baseline (Figure 7 and Table 10). Among treatment cohorts within each time point, the plasma agmatine levels were highest (*p* < 0.05) on the 15th, followed by the 1st and 30th day of agmatine and the 15th day of control administration at 30 and 60 min. They were also higher (*p* < 0.01) on the 1st and 15th day of agmatine versus the 15th day of control administration at 120 min. By 5 h after agmatine consumption, plasma agmatine concentrations were higher (*p* < 0.01) than after control consumption on the 15th day of administration. Plasma agmatine levels 24 h after consumption were higher (*p* < 0.05) on the 1st and 15th compared to the 30th day of agmatine administration and the 15th of control administration. The 15th-day agmatine Cmax was higher (*p* = 0.0464) for agmatine than the control administration (Table 11).

**Figure 8:**
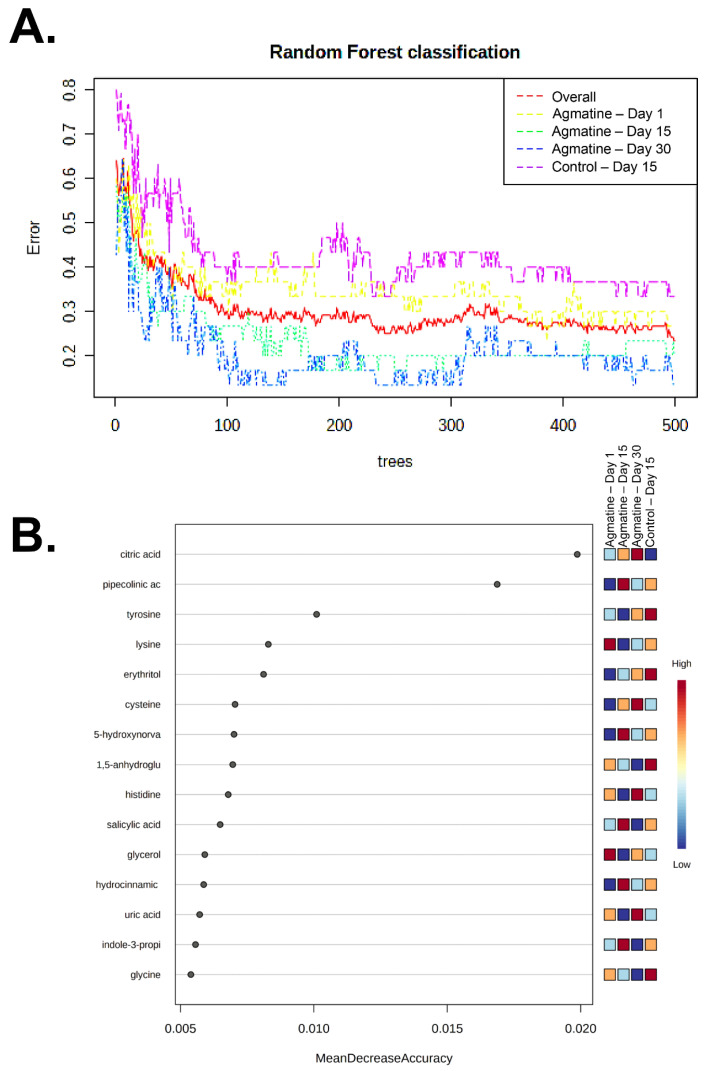
Random forest classification of treatment/time cohorts (agmatine 1st, 15th, 30th day; control 15th day). The total classification error rates of 500 trees are presented for overall (red), 1 (yellow), 15 (green), 30 (blue) days of agmatine and 15 (purple) days of control administration (**A**). A variable importance plot of the association of measured metabolites with each classifying treatment cohort (**B**). The metabolites are presented in decreasing importance, from the highest to the lowest point of the graph.

The effects of treatment and post-oral administration time were significant for 4 and 9 metabolites, respectively. There were interactions between treatment and post-administration time effects for one metabolite, citric acid (Table 12). The treatment effects on plasma citric acid concentration had the lowest *p*-value. The random forest for treatment classification using all samples (n = 120) identified 103 metabolites. The overall out-of-bag error rate, a measure of treatment cohort prediction accuracy, was 0.23. Classification errors for the 1st, 15th, and 30th day of agmatine administration and the 15th day of control administration were 0.27, 0.20, 0.133 and 0.33, respectively (Figure 8A, Table 13). Based on a mean decrease in prediction accuracy, the most important metabolite for classification accuracy was citric acid (Figure 8B). Citric acid plasma levels increased with the duration of agmatine administration.

## 4. Discussion

Based on the study results, all three hypotheses were accepted. The findings support that agmatine is bioavailable in horses following oral administration. The thoracic limb braking force is also higher following agmatine versus phenylbutazone administration at an agmatine dose reported to be effective for canine and human musculoskeletal pain [15,27]. Additionally, elevated plasma levels of citric acid after 15 days of agmatine administration may support reduced joint pain, potentially via anti-inflammatory effects and restoration of impaired citric acid cycle flux in neuropathic nerves [42,43]. Taken together, the investigative outcomes support the value of agmatine as an adjunct therapy to short-term phenylbutazone for equine thoracic limb OA discomfort.

Improved AAEP lameness scores after 14 and 29 days and increased peak vertical force after 14 days of phenylbutazone administration in this study are consistent with reported lameness reduction in horses with OA after 14 versus 7 days of the drug and this suggests that 2 weeks of administration may be necessary for the greatest impact [44]. However, phenylbutazone is reported to have significant gastrointestinal adverse effects after as little as 9 days of administration [45]. It is additionally reported to reduce equine articular cartilage proteoglycan synthesis after 14 days [46]. As such, administration of phenylbutazone for more than 14 days at a time, especially in horses with OA, is not generally recommended [47]. The lack of an effect of phenylbutazone after 29 days of administration in this study also suggests the development of drug tolerance. While this may not be well documented in horses, the development of anti-nociceptive tolerance to NSAIDs is documented in several species [48,49], and after 4 days of diclofenac, ketorolac, and lornoxicam administration in a rat nociceptive pain model [48]. Additionally, a recent meta-analysis of NSAIDs use by human knee OA patients revealed moderate pain reduction that peaked at 2 weeks, followed by diminished efficacy over 26 weeks [49]. It is possible that the prolonged use of phenylbutazone upregulated metabolism and elimination of the drug, both of which are faster in horses compared to many other species [50].

In quadrupeds, braking forces are highest in the thoracic limbs, in contrast to the higher pelvic limb propulsion forces [51,52]. Peak force represents a single point over the stance cycle, while impulse represents the area under the force–time curve or limb use over the entire stance. The two measures are complimentary but not identical and together provide more comprehensive information than either alone [53]. The greater improvements in braking impulse with agmatine after 14 days correspond to more sustained use of the limb for braking during stance, while higher braking force after 29 days indicates higher levels of limb use for braking [27]. The duration of agmatine effectiveness was previously reported for human disc-associated radiculopathy pain, when improvements on the visual analog pain scale continued for up to 80 days, although oral agmatine was discontinued after 14 days. Furthermore, improved limb function for up to 30 days was reported for dogs with mild coxofemoral joint OA who were administered agmatine daily [15,27,54]. Based on the results, the daily administration of agmatine appears to have a positive impact on braking ground reaction forces in equine thoracic limbs affected with OA, detectable after 14 and up to 30 days.

In addition to gait impacts, agmatine administration significantly reduced the glandular mucosa ulcer number and severity compared to phenylbutazone. The increase in the glandular mucosa ulcer severity score with phenylbutazone administration is consistent with established knowledge that ulceration occurs as a result of lost protective mechanisms versus acidic injury that tends to predominate in non-glandular mucosa ulceration [45,55,56]. It is possible that the study stall confinement may have contributed to some gastric ulceration. However, this was not evident in the study results, and stall confinement typically results in non-glandular gastric ulceration by increasing gastric acidity from intermittent feed deprivation [56,57]. As previously mentioned, agmatine’s gastroprotective effects have been thoroughly investigated in rodent models [30,31] and are reported to result from a number of cell signaling pathways, receptors, and growth factors [30,31,33,34]. Oral administration is also thought to have a direct acid-neutralizing effect from agmatine and its polyamine metabolites [58]. Results of this study indicate that oral agmatine administration does not appear to increase, and may reduce, existing equine glandular mucosa ulceration; however, further research is necessary before considering the compound for treatment or prevention.

Plasma agmatine quantification confirmed Cmax 30–60 min after oral administration in horses, similar to that in mice [59]. However, Cmax changed with the length of administration in this study, with the highest Cmax and AUC on the 15th versus the 1st and 30th days. Increases in Cmax and AUC after multiple oral doses are thought to indicate steady-state plasma levels when metabolizing capacity becomes saturated [60]. This has recently been reported for horses following oral curcumin administration when the compound was only detectable in plasma after 14 days of administration [61]. A drop in plasma agmatine levels by 30 days of administration may also indicate upregulation of metabolizing capacity or changes in distribution volume. In a murine study of oral agmatine administration, agmatine concentrations increased in plasma and the hippocampus after 7 days, but by 15 weeks, agmatine was undetectable in plasma, although the concentration increased in multiple brain regions [59]. Oral agmatine administration may require 15 days to reach a steady state, and subsequent increases in metabolism by 30 days may be responsible for lower plasma levels.

Based on the information above and the changes in the agmatine metabolite profile over the course of administration, it is plausible that agmatine metabolites, rather than agmatine itself, may be responsible for pain effects. Citrate was the metabolite most significantly affected by treatment in this study, and plasma levels of the metabolite increased with the duration of agmatine administration. Increased hepatic citrate levels were previously reported after murine oral agmatine administration, though the increase did not reach significance after 28 days [62]. Plasma citric acid may affect pain through anti-neuropathic effects, specifically via increased flux (activity) of the citric acid cycle within damaged nerves. In a murine model of diabetic-induced peripheral neuropathy, there were decreases in citric acid cycle metabolites, citric acid, ketoglutaric acid, succinic acid, fumaric acid, and malic acid, in the sciatic nerve that were attributed to inhibition of the cycle [42]. Additionally, citric acid, a well-known antioxidant, was demonstrated to minimize lipopolysaccharide-induced lipid peroxidation, inflammation, and NO synthase expression in mice [43]. Collectively, effects similar to these may reduce pain associated with OA in horses. However, determining the distinct effects and plasma levels of agmatine and agmatine metabolites at different agmatine dosing regimens and administration periods will be important to optimize potential therapeutic benefits.

Although the effects of increased plasma citric acid on OA were not measured in the present study, it is possible that higher levels resulting from agmatine consumption are protective of articular cartilage. Increased citric acid cycle flux, a primary metabolomic change associated with metformin, is postulated to have positive effects on human articular cartilage [63]. Metformin decreased the risk for total knee replacement in human patients with a body mass index of >30 kg/m^2^ (*p* = 0.11) [64] and the rate of joint replacement in those with type II diabetes mellitus [65]. Additionally, increased plasma citric acid levels may downregulate the degradation of arginine, a primary substrate for the citric acid cycle, via negative feedback mechanisms [66]. Arginine depletion is reported to be the most significant metabolomic change in human knee OA patients compared to those without OA in any joint [67]. The potential for a protective effect of agmatine on equine articular cartilage is appealing, but it requires substantially more investigation prior to any conclusions.

There are several acknowledged limitations to the study reported here. The small sample size could have negatively affected study power and led to type II errors despite steps to limit intra- and inter-animal effects. The number of participants was based on a power analysis using data from horse studies of phenylbutazone effects on equine limb use [68,69]. Steps to limit the outside effects also included the use of a crossover study design and outcome measures normalized to the baseline values of each animal as individual internal controls. Notably, differences among treatment groups in subjective gait measures did not reach statistical significance, unlike several objective gait measures. This was previously reported when intra-articular injection of lipopolysaccharide in horses resulted in a significant decrease in GRFs consistent with pain-related gait accommodations that were not detectable in subjective lameness scores [70]. This might also be attributable, in part, to a granularity of continuous numeric GRF data compared to ordinal AAEP lameness scale data. Nonetheless, the potential for type II errors cannot be fully ruled out. The 21-day washout period was longer than the 14 days washout periods reported for equine crossover studies that include phenylbutazone [71,72] and for a canine crossover study with agmatine, in which baseline GRF outcome measures did not differ among treatment blocks [27]. However, there is a potential for a carryover effect of agmatine into the successive treatment phase since there is limited available information about the pharmacokinetics of oral agmatine in mammals. Hence, outcomes should be considered specific to the research herd horse population and, in light of the acknowledged study limitations, until the effects of agmatine on limb use for naturally occurring OA are evaluated in a broader population.

## 5. Conclusions

This study confirms that agmatine is bioavailable via oral administration in horses, increases braking forces of equine thoracic limbs with OA, and appears to provide gastroprotection. Effects may be mediated directly by agmatine and indirectly by agmatine metabolites. Oral administration of agmatine may be an appealing addition to current strategies for the management of signs associated with equine OA.

## Figures and Tables

**Table 1:** American Association of Equine Practitioners Lameness Scale [35].

Grade	Clinical Signs
0	Lameness not perceptible under any circumstances.
1	Lameness is difficult to observe and is not consistently apparent, regardless of circumstances (e.g., under saddle, circling, inclines, hard surface, etc.).
2	Lameness is difficult to observe at a walk or when trotting in a straight line but consistently apparent under certain circumstances (e.g., weight-carrying, circling, inclines, hard surface, etc.).
3	Lameness is consistently observable at a trot under all circumstances.
4	Lameness is obvious at a walk.
5	Lameness produces minimal weight bearing in motion and/or at rest or a complete inability to move.

**Table 2:** EGUS Scale [39].

Grade	Signs
0	The epithelium is intact and there is no appearance of hyperaemia (reddening) or hyperkeratosis (yellow appearance to the squamous mucosa)
1	The mucosa is intact, but there are areas of reddening or hyperkeratosis (squamous)
2	Small, single, or multifocal lesions
3	Large, single, or multifocal lesions or extensive superficial lesions
4	Extensive lesions with areas of apparent deep ulceration

**Table 3:** Ulcer Number and Severity Scale [40].

Lesion Number Score	Signs
0	No lesions
1	1–2 localised lesions
2	3–5 localised lesions
3	6–10 lesions
4	>10 lesions or diffuse (or very large) lesions
**Lesion Severity Score**	**Signs**
0	No lesion
1	Appears superficial (only mucosa missing)
2	Deeper structures involved (greater depth than No. 1)
3	Multiple lesions and variable severity (1, 2 and/or 4)
4	Same as 2 and has active appearance (active = hyperaemic and/or darkened lesion crater)
5	Same as 4 plus active haemorrhage or adherent blood clot

**Table 4:** Changes in subjective AAEP Lameness Score. Min: minimum; Max: maximum; KS: *p*-value of Kolmogorov–Smirnov test.

	14 Days					29 Days				
Treatment	LS Mean	SEM	Min	Max	KS	LS Mean	SEM	Min	Max	KS
Agmatine	−0.7060	0.6311	−3	1	>0.1000	−0.8060	0.6311	−2	0	>0.1000
Phenylbutazone	−1.7500	0.5729	−5	−0.5	0.0285	−2.0833	0.5729	−5	−1	0.0171
Control	−2.2431	0.7184	−4	−1	>0.1000	−1.3060	0.6311	−3	0	>0.1000

**Table 5:** Fixed effects parameter estimates of subjective lameness score changes. Std Error: standard error; Prob > |t|: *p*-value; 95% Lower: 95% confidence interval lower limit; 95% Upper: 95% confidence interval upper limit.

Term	Estimate	Std Error	Prob > |t|	95% Lower	95% Upper
Intercept	−1.4824	0.2500	0.0032	−2.1584	−0.8064
Treatment[Agmatine]	0.7264	0.3652	0.0599	−0.0331	1.4859
Treatment[Phenylbutazone]	−0.4343	0.3512	0.2292	−1.1621	0.2936
Time[14.00]	−0.0840	0.2574	0.7476	−0.6198	0.4518
Treatment[Agmatine] × Time[14.00]	0.1340	0.3646	0.7171	−0.6254	0.8933
Treatment[Phenylbutazone] × Time[14.00]	0.2506	0.3491	0.4808	−0.4762	0.9775

**Table 6:** Fixed effects parameter estimates of peak force percent changes. Std Error: standard error; Prob > |t|: *p*-value; 95% Lower: 95% confidence interval lower limit; 95% Upper: 95% confidence interval upper limit.

Term	Estimate	Std Error	Prob > |t|	95% Lower	95% Upper
Fz					
Intercept	5.2995	1.7762	0.0266	0.8783	9.7208
Treatment[Agmatine]	−3.2440	0.8066	<.0001	−4.8314	−1.6566
Treatment[Phenylbutazone]	4.8309	0.7871	<.0001	3.2819	6.3799
Time[29-14]	−0.5435	0.7904	0.4922	−2.0990	1.0120
Treatment[Agmatine] × Time[29-14]	2.0362	1.1218	0.0705	−0.1715	4.2439
Treatment[Phenylbutazone] × Time[29-14]	−2.6909	1.0788	0.0132	−4.8139	−0.5678
Fy(b)					
Intercept	3.8280	2.5372	0.1755	−2.1831	9.8392
Treatment[Agmatine]	1.9557	1.7874	0.2748	−1.5625	5.4738
Treatment[Phenylbutazone]	−2.9649	1.7903	0.0988	−6.4887	0.5589
Time[29-14]	−1.7619	1.7704	0.3205	−5.2467	1.7229
Treatment[Agmatine] × Time[29-14]	3.9358	2.5003	0.1166	−0.9859	8.8575
Treatment[Phenylbutazone] × Time[29-14]	−2.7610	2.4450	0.2598	−7.5738	2.0518
Fy(p)					
Intercept	11.2977	2.7057	0.0041	4.9019	17.6934
Treatment[Agmatine]	−3.2127	2.2398	0.1527	−7.6232	1.1978
Treatment[Phenylbutazone]	1.1749	2.2657	0.6045	−3.2866	5.6364
Time[29-14]	1.7842	2.2742	0.4334	−2.6942	6.2626
Treatment[Agmatine] × Time[29-14]	6.7091	3.1965	0.0368	0.4144	13.0037
Treatment[Phenylbutazone] × Time[29-14]	−2.0963	3.1324	0.5040	−8.2648	4.0722

**Table 7:** Fixed effect parameter estimates of on-impulse percentage changes. Std Error: standard error; Prob > |t|: *p*-value; 95% Lower: 95% confidence interval lower limit; 95% Upper: 95% confidence interval upper limit.

Term	Estimate	Std Error	Prob > |t|	95% Lower	95% Upper
Fz					
Intercept	1.7136	1.4031	0.2735	−1.8330	5.2603
Treatment[Agmatine]	0.5895	0.5667	0.2991	−0.5258	1.7048
Treatment[Phenylbutazone]	−0.0043	0.5531	0.9939	−1.0928	1.0843
Time[29-14]	0.1401	0.5553	0.8010	−0.9527	1.2328
Treatment[Agmatine] × Time[29-14]	−2.4104	0.7881	0.0024	−3.9614	−0.8594
Treatment[Phenylbutazone] × Time[29-14]	1.2398	0.7579	0.1029	−0.2517	2.7314
Fy(b)					
Term	Estimate	Std Error	Prob>|t|	95% Lower	95% Upper
Intercept	0.4045	1.8703	0.8323	−3.6534	4.4625
Treatment[Agmatine]	5.7723	2.3726	0.0156	1.1020	10.4427
Treatment[Phenylbutazone]	−8.0360	2.3506	0.0007	−12.6638	−3.4081
Time[29-14]	−1.3715	2.3564	0.5610	−6.0102	3.2671
Treatment[Agmatine] × Time[29-14]	−4.0846	3.3197	0.2196	−10.6194	2.4502
Treatment[Phenylbutazone] × Time[29-14]	3.5902	3.2689	0.2730	−2.8445	10.0249
Fy(p)					
Intercept	6.1930	2.5884	0.0378	0.4263	11.9598
Treatment[Agmatine]	−1.9878	2.6589	0.4554	−7.2255	3.2499
Treatment[Phenylbutazone]	0.6818	2.6312	0.7958	−4.5017	5.8653
Time[29-14]	3.9900	2.6660	0.1358	−1.2620	9.2419
Treatment[Agmatine] × Time[29-14]	1.1710	3.7293	0.7538	−6.1758	8.5177
Treatment[Phenylbutazone] × Time[29-14]	2.7214	3.6295	0.4541	−4.4286	9.8714

**Table 8:** Changes in gastric ulceration score. Min: minimum; Max: maximum; KS: *p*-value of Kolmogorov–Smirnov test.

Treatment	LS Mean	SEM	Min	Max	KS
EGUS					
Agmatine	−0.2000	0.5397	−2	2	>0.1000
Phenylbutazone	0.6667	0.4927	0	2	>0.1000
Control	−0.8000	0.5397	−2	1	>0.1000
Number (Nonglandular)					
Agmatine	0.2000	0.6794	−2	3	0.0525
Phenylbutazone	1.0000	0.6202	0	3	>0.1000
Control	−0.6000	0.6794	−3	1	>0.1000
Severity (Nonglandular)					
Agmatine	−0.4000	0.6887	−2	2	>0.1000
Phenylbutazone	1.1667	0.6287	0	4	>0.1000
Control	−0.2000	0.6887	−2	2	>0.1000
Number (Glandular)					
Agmatine	−1.6000	0.5805	−4	0	>0.1000
Phenylbutazone	0.5000	0.5299	0	1	0.0557
Control	−0.4000	0.5805	−2	2	>0.1000
Severity (Glandular)					
Agmatine	−1.2000	0.4374	−2	0	0.0261
Phenylbutazone	0.8333	0.3993	0	2	>0.1000
Control	0.2000	0.4374	−1	2	0.0222

**Table 9:** Fixed effects parameter estimates of changes in gastric ulceration score. Std Error: standard error; Prob > |t|: *p*-value; 95% Lower: 95% confidence interval lower limit; 95% Upper: 95% confidence interval upper limit.

Term	Estimate	Std Error	Prob > |t|	95% Lower	95% Upper
EGUS					
Intercept	−0.1111	0.3028	0.7196	−0.7653	0.5431
Treatment[Agmatine]	−0.0889	0.4345	0.8411	−1.0276	0.8498
Treatment[Phenylbutazone]	0.7778	0.4155	0.0839	−0.1198	1.6753
Number (Nonglandular)					
Intercept	0.2000	0.3812	0.6086	−0.6235	1.0235
Treatment[Agmatine]	0.0000	0.5469	1.0000	−1.1816	1.1816
Treatment[Phenylbutazone]	0.8000	0.5230	0.1501	−0.3298	1.9298
Severity (Nonglandular)					
Intercept	0.1889	0.3864	0.6331	−0.6460	1.0237
Treatment[Agmatine]	−0.5889	0.5545	0.3075	−1.7868	0.6090
Treatment[Phenylbutazone]	0.9778	0.5302	0.0881	−0.1676	2.1232
Number (Glandular)					
Intercept	−0.5000	0.3257	0.1487	−1.2036	0.2036
Treatment[Agmatine]	−1.1000	0.4673	0.0350	−2.1096	−0.0904
Treatment[Phenylbutazone]	1.0000	0.4468	0.0434	0.0347	1.9653
Severity (Glandular)					
Intercept	−0.0556	0.2454	0.8244	−0.5857	0.4746
Treatment[Agmatine]	−1.1444	0.3521	0.0063	−1.9051	−0.3838
Treatment[Phenylbutazone]	0.8889	0.3367	0.0204	0.1615	1.6162

**Table 10:** Plasma agmatine levels. Mean and standard error of the mean (SEM) of plasma agmatine levels (ng/mL) for each treatment (agmatine 1st, 15th, 30th day; control 15th day) and time (0, 30, 60, 120 min, 5, 24 h). KS: *p* value of Kolmogorov–Smirnov test.

Time (min)	0		30		60		120		300		1440		
	Mean	SEM	Mean	SEM	Mean	SEM	Mean	SEM	Mean	SEM	Mean	SEM	KS
Agmatine 1 day	8.1800	2.1974	24.8600	8.0955	25.8800	8.1512	23.1000	7.4267	13.5800	1.8699	20.7800	4.8687	>0.1000
Agmatine 15 day	7.0400	2.0653	39.2200	9.4204	41.2200	11.3124	25.7800	8.2086	22.4800	8.5319	20.2600	9.3353	>0.1000
Agmatine 30 day	6.4600	1.2488	26.3800	6.9356	18.8600	4.3970	13.2400	2.1407	13.4800	2.6973	6.3200	1.9765	>0.1000
Control 15 day	3.5600	0.6274	10.1000	2.8327	8.3800	2.0001	6.8800	2.0793	6.3400	2.2482	7.1400	2.2825	>0.1000

**Table 11:** Equine Plasma Agmatine Pharmacokinetic Parameters. The peak plasma concentration (Cmax), time to reach Cmax (Tmax), and area under the agmatine plasma concentration versus time curve (AUC) (mean ± SEM) for each treatment (agmatine 1st, 15th, 30th day; control 15th day). An asterisk indicates a significant difference from control 15th-day levels (*p* < 0.05).

Treatment	Cmax (ng/mL)	Tmax (h)	AUC (ng/mL h)
Agmatine 1 day	29.9 ± 7.5	10.4 ± 5.6	426.9 ± 84
Agmatine 15 day	43.5 ± 11.2 *	1.5 ± 0.9	543.6 ± 206.4
Agmatine 30 day	27 ± 6.8	0.6 ± 0.1	263.8 ± 47.3
Control 15 day	12.1 ± 2.5	6.5 ± 5.8	163.6 ± 40.6

**Table 12:** Effects of Time or Treatment on Equine Agmatine Plasma Metabolite Levels. Adjusted *p*-values (adj. *p*) for the effects of treatment (agmatine 1st, 15th, 30th days; control 15th day) and time (0, 30, 60, 120, minutes, 5, 24 h) on plasma levels of the indicated agmatine metabolites.

Metabolite	Treatment (adj. *p*)	Time (adj. *p*)
Citric acid	3.05 × 10^−5^	0.05334
3-hydroxybenzoic acid	0.0090779	0.023406
Cysteine	0.016639	0.47485
Glucose	0.019604	0.071763
Glycerol	0.039944	0.5698
3-hydroxybutyric acid	0.11369	0.022329
Hippuric acid	0.11732	0.0072771
Salicylic acid	0.18872	0.010535
Aconitic acid	0.36333	0.0223
Xylulose	0.36333	0.030289
Xylose	0.48526	0.010535
Linolenic acid	0.53124	0.00055627
Oleic acid	0.53955	0.00055627
Asparagine	0.67783	0.0012954

**Table 13:** Confusion Matrix for a Random Forest Prediction Model of Treatment and Duration. The misclassification rate (class error) is shown for the predicted treatment cohorts (columns) versus the actual treatment cohorts (rows).

N = 120	Predicted					
**Actual**		**Agmatine—Day 1**	**Agmatine—Day 15**	**Agmatine—Day 30**	**Control—Day 15**	**Class Error**
	Agmatine—Day 1	22	3	2	3	0.267
	Agmatine—Day 15	1	24	4	1	0.2
	Agmatine—Day 30	2	1	26	1	0.133
	Control—Day 15	3	5	2	20	0.333

## Data Availability

The datasets supporting the conclusions of this article are included within the article and its additional files. All datasets used and analyzed during the current study are available from the corresponding author upon reasonable request.
